# Declining Coral Skeletal Extension for Forereef Colonies of *Siderastrea siderea* on the Mesoamerican Barrier Reef System, Southern Belize

**DOI:** 10.1371/journal.pone.0014615

**Published:** 2011-02-16

**Authors:** Karl D. Castillo, Justin B. Ries, Jack M. Weiss

**Affiliations:** 1 Department of Marine Sciences, University of North Carolina at Chapel Hill, Chapel Hill, North Carolina, United States of America; 2 Curriculum for the Environment and Ecology, University of North Carolina at Chapel Hill, Chapel Hill, North Carolina, United States of America; California Academy of Sciences, United States of America

## Abstract

**Background:**

Natural and anthropogenic stressors are predicted to have increasingly negative impacts on coral reefs. Understanding how these environmental stressors have impacted coral skeletal growth should improve our ability to predict how they may affect coral reefs in the future. We investigated century-scale variations in skeletal extension for the slow-growing massive scleractinian coral *Siderastrea siderea* inhabiting the forereef, backreef, and nearshore reefs of the Mesoamerican Barrier Reef System (MBRS) in the western Caribbean Sea.

**Methodology/Principal Findings:**

Thirteen *S. siderea* cores were extracted, slabbed, and X-rayed. Annual skeletal extension was estimated from adjacent low- and high-density growth bands. Since the early 1900s, forereef *S. siderea* colonies have shifted from exhibiting the fastest to the slowest *average annual skeletal extension*, while values for backreef and nearshore colonies have remained relatively constant. The *rates of change in annual skeletal extension* were −0.020±0.005, 0.011±0.006, and −0.008±0.006 mm yr^−1^ per year [mean±SE] for forereef, backreef, and nearshore colonies respectively. These values for forereef and nearshore *S. siderea* were significantly lower by 0.031±0.008 and by 0.019±0.009 mm yr^−1^ per year, respectively, than for backreef colonies. However, only forereef *S. siderea* exhibited a statistically significant decline in annual skeletal extension over the last century.

**Conclusions/Significance:**

Our results suggest that forereef *S. siderea* colonies are more susceptible to environmental stress than backreef and nearshore counterparts, which may have historically been exposed to higher natural baseline stressors. Alternatively, sediment plumes, nutrients, and pollution originating from watersheds of Guatemala and Honduras may disproportionately impact the forereef environment of the MBRS. We are presently reconstructing the history of environmental stressors that have impacted the MBRS to constrain the cause(s) of the observed reductions in coral skeletal growth. This should improve our ability to predict and potentially mitigate the effects of future environmental stressors on coral reef ecosystems.

## Introduction

Coral reefs around the world are threatened by global warming [1], [2], increased sedimentation [3], [4], eutrophication [5], overfishing [6], disease [7], [8], ocean acidification [2], [9], and other natural and anthropogenic stressors. In recent decades, the health of Caribbean corals has declined dramatically [6], [10], with average hard coral cover on some reef communities declining by 80% [10]. The Mesoamerican Barrier Reef System (MBRS) extends over 1000 km along portions of the Atlantic coast of Mexico, Honduras, and Guatemala, and along the entire coast of Belize. Over the last two decades, the combined human population in Belize, Guatemala, and Honduras has increased from 13.2 to 22.3 million, an increase of approximately 69% (Table S1) [11]. The number of people within these three countries inhabiting watersheds that drain into the Caribbean Sea exceeded 13 million by mid-2010 (Table S1). Global warming, increased environmental stress resulting from expanding coastal populations, and changes in regional land use have already negatively impacted the MBRS [12]. These negative impacts are expected to escalate as these regions continue to develop [12]. Understanding how environmental changes have impacted coral reefs throughout the recent past should assist us in predicting how continued changes will affect reefs in the future.

Coral skeletal extension estimated from the width of coupled high and low density annual growth bands within coral cores have been interpreted as an indicator of coral health and ecological success [13], [14]. They may also reflect the health of the greater reef system, since many reef dwelling organisms depend on the complex reef structure built from the aragonite skeletons of corals [15]. Annual skeletal extension has been quantified for several coral species on reef ecosystems around the world [13], [16]–[19], and point to a global decline in the rate of coral skeletal extension [15], [20]–[22]. Some studies, however, have also documented increasing skeletal extension for coral species of the genus *Porites* throughout the recent past [16], [23].

The massive zooxanthellate scleractinian coral *Siderastrea siderea* is one of the primary reef-building corals of the Caribbean Sea [24]. It inhabits shallow-to-moderate depth reef environments and can grow to a diameter of more than a meter. *S. siderea* is well-suited for investigating long-term trends in skeletal extension because it grows relatively slowly and because colonies are generally long-lived [25]. Therefore, even short cores (<1 m) can record information reflecting relatively long intervals (∼100 yrs) of environmental change. *S. siderea* is also a hardy species capable of surviving severe environmental stress, which results in relatively uninterrupted growth histories within cores extracted from these corals [25]. Despite the suitability of *S. siderea* for coral coring studies, as well as its important reef-building role on many Caribbean reefs, *S. siderea* has been relatively underutilized as an archive of environmental change and coral health.

In this study, we investigate variations in the skeletal extension of *S. siderea* colonies from across an inshore-offshore gradient of the MBRS in southern Belize. Specifically, we quantified *average annual skeletal extension* and *rates of change in annual skeletal extension* for forereef, backreef, and nearshore *S. siderea* colonies, and examined whether rates of change in annual skeletal extension differed amongst these three reef zones, as well as from zero, over the last century. Establishing and comparing the history of skeletal extension for *S. siderea* across this inshore-offshore gradient enables us to infer whether colonies of *S. siderea* from different reef zones of the MBRS exhibit differing vulnerabilities to environmental stressors. This should improve our understanding of how corals from different regions of the MBRS responded to environmental stressors over the last century and how they are likely to respond in the future.

In general, shallow forereef corals (located on the oceanic side of the reef crest) are exposed to high wave activity [26] and are generally stenothermal, i.e., residing in cooler, more thermally stable seawater [27], [28]. In contrast, backreef habitats are located on the shoreward side of the reef crest and are characteristically shallower and have more restricted circulation. This maintains a more eurythermal environment with relatively warmer and more variable seawater temperatures than forereef environments [28]. Nearshore reef habitats generally consist of patch reefs that are more proximal (<10 km) to the coast and, therefore, to the source of anthropogenic stress. Corals in these nearshore habitats are typically exposed to higher rates of fluvial sedimentation, to more concentrated pollution from runoff, and to greater seasonality in ambient seawater temperatures [28], [29]. Thus, a natural stress gradient exists for corals from the nearshore (most stressful) to the forereef (least stressful) habitats. This gradient is thought to cause physiological, and potentially, genetic differences amongst coral populations across reef zones [28], [30], [31].

The coral *S. siderea* was investigated in the present study because it is well represented in each of these three sub-environments of the MBRS. Because nearshore and backreef colonies have been historically exposed to more regular and more intense environmental stressors, they are potentially more acclimatized and/or adapted to these stressors than forereef colonies [28], [30], [31]. We therefore hypothesize that nearshore and backreef colonies of *S. siderea* have exhibited greater resistance to increasing environmental stress over the past century than forereef colonies. We evaluate this hypothesis by reconstructing and comparing the history of skeletal extension of *S. siderea* corals inhabiting these three reef zones of the MBRS. Identifying systematic differences in temporal patterns of coral skeletal extension amongst these three reef zones should improve our ability to assess which regions of the MBRS, and potentially other reef systems, are most vulnerable to future increases in natural and anthropogenic stress.

## Methods

### Extraction of coral cores

**Figure 1:**
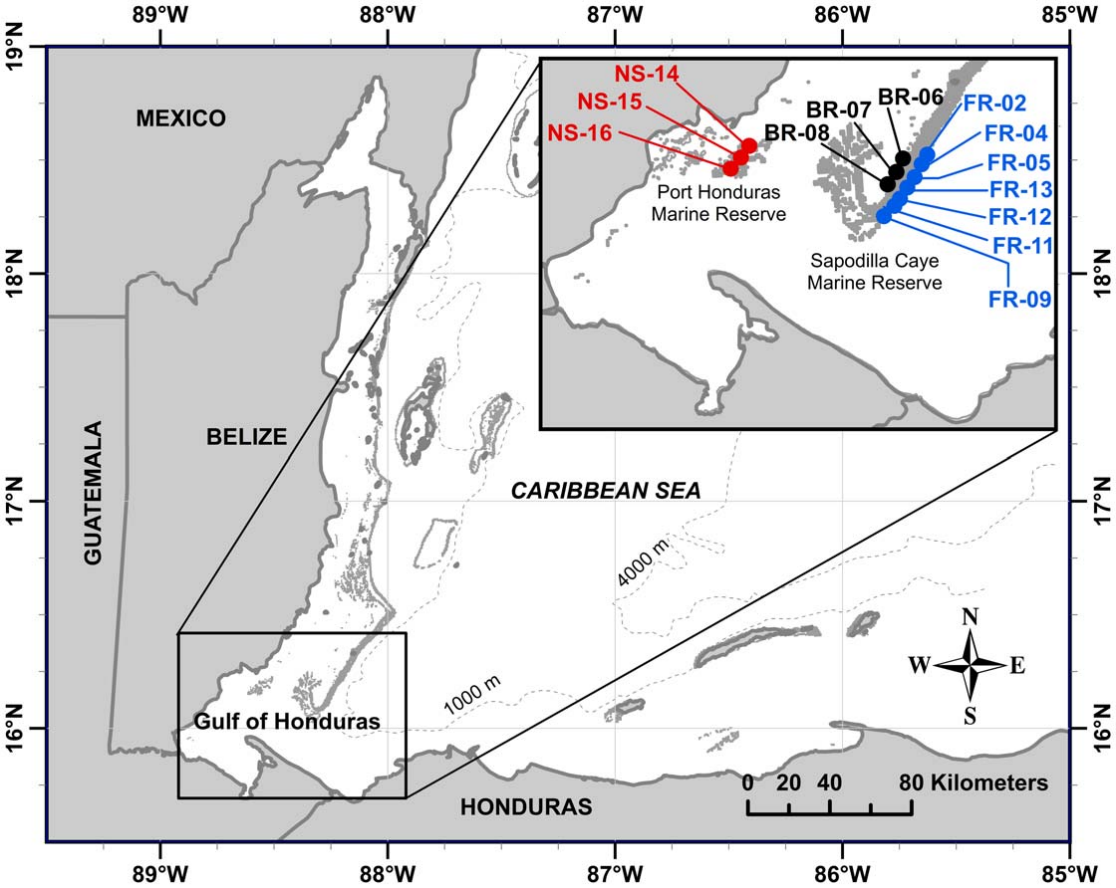
Map of coral core extraction sites on the Mesoamerican Reef. Forereef coring sites include FR-02, FR-04, FR-05, FR-09 and FR-11 through FR-13. Backreef coring sites include BR-06 through BR-08. Nearshore reef coring sites include NS-14 through NS-16. Nearshore sites are ∼30 to 40 km from backreef and forereef sites. Backreef coring sites are separated from forereef sites by the reef crest over distances ranging from ∼1.0 to 4.5 km.

Cores were extracted from colonies of *S. siderea* from the forereef, backreef, and nearshore reef zones of the MBRS in southern Belize. Forereef and backreef coral cores were obtained from the Sapodilla Cayes Marine Reserve, on the seaward and shoreward side of the reef crest, respectively. Nearshore coral cores were obtained from within 10 km of the coast in the Port Honduras Marine Reserve (Fig. 1). Collection permits were obtained from the Belize Fisheries Department, and all cores were collected and transported pursuant to local, federal, and international regulations.

A total of thirteen cores were extracted from thirteen different colonies of *S. siderea* in February of 2009. Seven cores were extracted from forereef colonies, 3 cores from backreef colonies, and 3 cores from nearshore colonies (Fig. 1, Table 1). Cores were extracted by SCUBA divers using a 2-horsepower hand-held pneumatic core drill (*CP 315; Chicago Pneumatic; Westfield, Massachusett*s) affixed with a hollow extension rod (5 cm in diameter, 90 cm in length) and a wet diamond core bit (5 cm in diameter, 30 cm in length). Compressed air from SCUBA cylinders located on a boat was used to power the pneumatic drill. Drilling each core required a total of 5 to 8 standard size SCUBA cylinders and approximately 30–45 minutes of continuous drilling. Coral cores, approximately 20 to 100 cm in length, and 5 cm in diameter, were collected from the center of each coral colony parallel to the coral's vertical growth axis. Cores were extracted from colonies that appeared healthy and were without any obvious abnormalities, scarring, bleaching, or disease. Coral samples were collected from colonies between depths of 4 and 5 m within each of the three reef zones. After extracting each core, a concrete plug was inserted into the drilled holes and sealed with *Z-spar* underwater epoxy.

**Table 1:** Number of cores and years of growth.

Reef Zone	Core #	Core ID	Years of Growth
Backreef (BR)	1	BR-06	80
	2	BR-07	83
	3	BR-08	19
Nearshore (NS)	1	NS-14	44
	2	NS-15	61
	3	NS-16	98
Forereef (FR)	1	FR-02	94
	2	FR-04	69
	3	FR-05	23
	4	FR-09	29
	5	FR-11	47
	6	FR-12	79
	7	FR-13	41

Number of cores extracted from each reef zone, core identification number, and years of growth (estimated by counting paired high-low density band annual cycles) for each coral core evaluated in the study.

### Determination of skeletal extension

Newly extracted coral cores were rinsed with 95% ethanol in the field, stored in 5-cm-diameter PVC tubes and transported to the University of North Carolina at Chapel Hill for analysis. Coral tissue from the surface of the cores was removed with a water pick (*Water Pik®)*. The top portions of the cores were thoroughly rinsed with 95% ethanol to eliminate any remaining coral tissue. Six-mm thick slabs were cut vertically from the center of each core using a water-cooled trim saw.

Coral slabs were air dried and X-rayed from a source-to-object distance of 100 cm at 6.0 mA s^−1^ and at 40 kV. This was performed with a *Fuji FCR (Fujifilm Medical Systems USA, Inc., Stamford Connecticut*) radiography system at the UNC-Chapel Hill Campus Health Services Radiology Department to reveal annual cycles in skeletal density.

**Figure 2:**
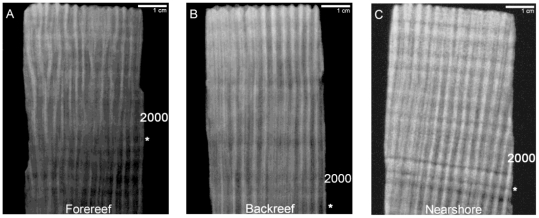
X-radiographs of sample coral cores. Core sections represent the most recent years of skeletal extension for *Siderastrea siderea* from the (A) forereef [core FR-12], (B) backreef [core BR-06], and (C) nearshore reef [core NS-14]. Numbers correspond to year of paired high-low density annual growth bands. Asterisks correspond to the annual growth bands formed during the 1998 coral bleaching event.

X-rayed films were digitized using a *Vidar VXR* film digitizer *(Vidar Systems Corporation, Herndon Virginia).* Digital X-radiographs were transferred into *Coral X-radiograph Densitometry System (Coral XDS v. 3.0)* [32] software for processing (Fig. 2). In general, *S. siderea* deposits low density bands approximately during the dry season (December – May) and high density bands approximately during the wet season (June – November) [25]. We used the extension/luminance mode and the half-range delimiting function (delimits bands based on the mean of adjacent maximum and minimum luminance) in *Coral XDS* to identify annual cycles in skeletal extension. This mode requires input of the coral X-radiograph image and the number of pixels per centimeter in the image [32], which was calculated for each core by measuring the actual distance between distinguishing features on the core slabs using a digital caliper and comparing it with the pixel distance between these features in the digital image.

The chronologies of the cores were established by identifying the most recent high density band deposited during the wet season of 2008, and counting backwards in time. We used visual cross-dating techniques to examine annual skeletal extension for cores within the same reef zone, since it is likely that these corals will share major growth patterns (as they are generally exposed to similar environmental conditions). Cores were visually cross-dated by identifying signature years of narrow growth to prevent dating errors associated with partial, missing, or double rings—a procedure commonly used in dendrochronology [33].

### Statistical analyses

**Figure 3:**
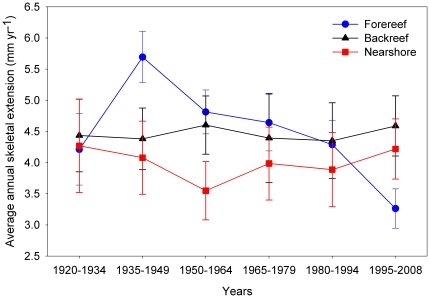
Average annual skeletal extension. Mean annual skeletal extension over 15-year intervals for *Siderastrea siderea* from the forereef (*n* = 7), backreef (*n* = 3), and nearshore (*n* = 3) reef environments of the Mesoamerican Barrier Reef System. Means were estimated using a random intercepts model. Error bars represent ±1 standard error.

As an initial exploration of the data we grouped observations from each core amongst the three reef zones into 15-year increments and estimated average annual skeletal extension using a random intercepts model to account for observational heterogeneity (Fig. 3). Rather than performing statistical analysis on a single master chronology from each reef zone, individual core chronologies were analyzed so as to address the hierarchical nature of the dataset. Annual skeletal extension within a core are inevitably highly correlated across time (i.e., not independent observations), but are approximately independent amongst different cores within the same reef zone. A linear regression of annual skeletal extension with time was achieved by fitting a set of mixed effects models that treated the individual core as a structural variable. A residual temporal correlation structure was employed to determine if the random effects adequately accounted for the correlation over time. To assess the need for random effects, the method of generalized least squares was employed to fit a corresponding set of models with residual correlation structures but without random effects. The use of mixed effects and time series methods to model coral skeletal extension correctly distinguishes observational units from sampling units, recognizes that sampling variation exists both within and between core time series, and addresses the temporal autocorrelation structure that is inherently present in such data. This approach also properly accounts for data imbalance—the fact that some cores provide a longer time series of annual skeletal extension than others [34]–[38].

Statistical analyses were carried out using the *nlme package* [39] of *R 2.9.0* [40], and *Proc Mixed of SAS/STAT ® software version 9.1 of SAS System for Windows* [41]. Rather than assessing and comparing average annual skeletal extension alone, as is commonly described in the literature, our goal was to also describe how rates of change in annual skeletal extension of *S. siderea* varied throughout the last century and amongst the forereef, backreef, and nearshore colonies. A sequence of models was fit to determine how best to describe the structural form of the data and to test the hypotheses of interest. Several models were tested, including (1) an ordinary regression model, (2) a random intercepts model with no predictors (i.e., an unconditional means model), (3) a random intercepts model that includes time as a predictor, (4) a random slopes and intercepts model in which the intercept and coefficient of time (slope) were allowed to be random, (5) a more complex version of model 3 and 4 that included level-2 predictors such as reef zone, (6) a version of model 5 that included a level-1 (residual) correlation structure, and (7) a version of model 6 that possessed a residual correlation structure without additional random effects.

The variable year was ‘centered’ using a centering constant of 1967 because this minimized correlation between the random slopes and intercepts. In general, centering enhances model interpretability and improves numerical stability by increasing the likelihood that the optimization algorithm converges on the correct solution. The estimate of the slope is unchanged by centering, but the intercept will estimate the mean value of the response variable in year 1967 (rather than in year zero of the uncentered model). The role of centering in mixed effects models is discussed in greater detail in O'Connor et al. (2007) [42].

Akaike Information Criterion (AIC) was used to identify the best-fit model [43]. AIC provides a measure of the explanatory power of a model discounted by the number of parameters that contributed to its construction; a lower value indicates a better fitting model. Of all the models that were fit, the random intercepts model with an ARMA (1, 1) correlation structure for the residuals yielded the lowest AIC. An ARMA (1, 1) correlation structure combines an autoregressive model of order 1 with a moving average model of order 1. The appropriateness of the ARMA (1, 1) structure was also indicated by patterns observed in plots of the autocorrelation and partial autocorrelation functions of the residuals. The final random intercepts model was
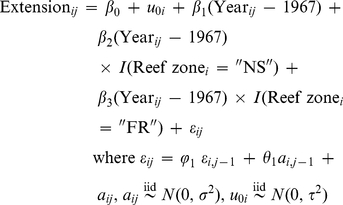
(1)


Here "*i*" references the core and "*j*" denotes an individual annual observation within that core. "*I*" is an indicator variable that takes on the value "1" if the condition in parentheses (Reef zone*_i_*  =  "XX") is true, and "0" if the condition is false. The construct "Reef zone" has been incorporated into the regression model as two dummy variables with "backreef" serving as the "reference group". The term "*u*_0*i*_" denotes the random intercept. It is constant for observations coming from the same core but differs for observations coming from different cores. Thus, equation (1) translates into three different fixed effects regression equations, one for each reef zone in equation (2) below, and thirteen different mixed effects equations, one for each core.
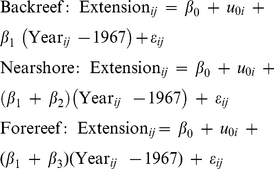
(2)


Here *β*_2_ and *β*_3_ measure the difference in rate of change in annual skeletal extension between the backreef coral colonies and the forereef and nearshore colonies. To investigate whether skeletal extension varied over time (i.e., slopes were equal to "zero" for *S. siderea* within the three different reef zones), estimates of the rates of change in skeletal extension derived from the ARMA (1, 1) random intercepts model along with the 50% and 95% confidence intervals were displayed in a plot popularized by Gelman and Hill (2006) [35]. These types of plots provide a range of uncertainty for the statistics presented.

Although index master chronologies were not used in developing the model presented here (i.e., analysis was done on actual annual skeletal extension of individual core time series), standardized annual skeletal extension has been displayed to facilitate comparison with coral skeletal extension reported elsewhere in the literature. Following standard protocol developed for dendrochronology [44], annual skeletal extension for each coral core was standardized by dividing yearly extension by mean annual skeletal extension calculated for the entire core.

## Results

### Differences in average skeletal extension amongst reef zones

Since the mid-1930's forereef colonies have shifted from exhibiting the fastest to the slowest average annual skeletal extension while values for backreef and nearshore *S. siderea* colonies have remained relatively consistent over this interval (Fig. 3).

### Differences in rates of change in annual skeletal extension over time

**Figure 4:**
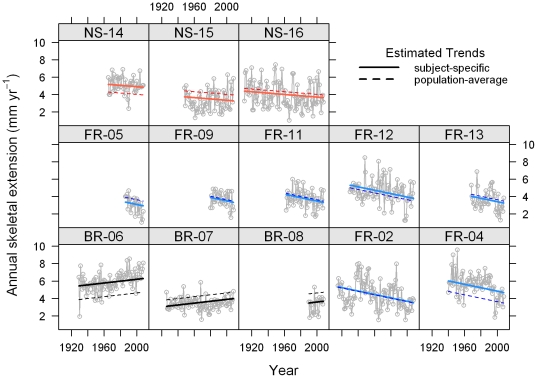
Trends in annual skeletal extension. Estimated trend lines for individual *Siderastrea siderea* cores from the forereef (FR), backreef (BR), and nearshore (NS) reef environments. Trend lines superimposed on time series plots are from the AIC-best temporal trend model, a random intercepts model in which an ARMA (1, 1) correlation structure was used for the residuals [equation (1)]. Dashed lines correspond to the population-average and solid lines correspond to subject-specific models. The subject-specific trend lines add predictions of the cores' random intercepts to the population-average model. This causes different cores from the same reef zone to have offset yet parallel trend lines.

**Figure 5:**
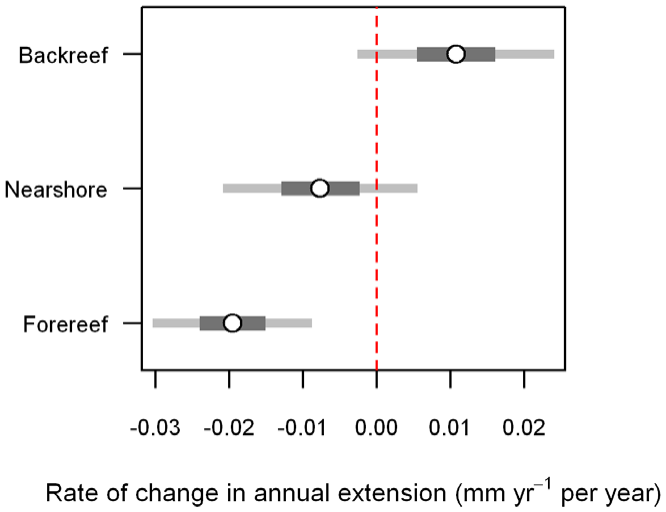
Regression analysis results for change in annual skeletal extension over time. 95% confidence intervals (light grey) and 50% confidence intervals (dark grey) for the model-derived average yearly change in annual skeletal extension for *Siderastrea siderea* corals from the forereef, backreef and nearshore reef environments. Interval estimates are from a random intercepts model with an ARMA (1, 1) correlation structure for the residuals [equation (1)].

Rates of change in annual skeletal extension for forereef *S. siderea* colonies were more negative than for backreef and nearshore colonies (Figs. 4, 5). The ARMA (1, 1) random intercepts model yielded the following three equations for the rates of change in annual skeletal extension.
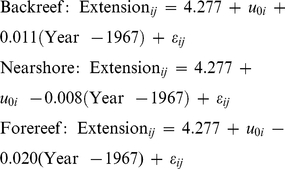
(3)


The rates of change in annual skeletal extension for backreef *S. siderea* colonies was assigned as the "reference group" in the ARMA (1, 1) random intercepts model. Thus, the rates of change in annual skeletal extension for forereef (−0.020±0.005 mm yr^−1^ per year) and nearshore (−0.008±0.006 mm yr^−1^ per year) *S. siderea* colonies were compared with that of the backreef colonies (+0.011±0.006 mm yr^−1^ per year; Fig. 4, 5). The rate of change in annual skeletal extension for forereef *S. siderea* was 0.031 (±0.008) mm yr^−1^ per year less than that for backreef *S. siderea* (*t* = −3.74, *p*<0.001). The rate of change in annual skeletal extension for nearshore *S. siderea* was 0.019 (±0.009) mm yr^−1^ per year less than that for backreef *S. siderea* (*t* = −2.06, *p* = 0.039; Table 2).

**Table 2:** Parameter estimates for the AIC-best model, equation (1), evaluating difference in rate of change in annual skeletal extension over approximately the last century for nearshore and forereef colonies relative to backreef colonies.

Parameter	Estimate	SE	*t-*statistic	*p-*value
Intercept: *β*_0_	4.277	0.242	17.67	<0.001
Year – 1967	0.011	0.006	1.65	0.099
Year –1967: Reef zone = NS: *β*_2_	−0.019	0.009	−2.06	0.039
Year –1967: Reef zone = FR: *β*_3_	−0.031	0.008	−3.74	<0.001
*σ* ^2^	1.430	—	—	—
*ϕ;*_1_	0.841	—	—	—
*θ*_1_	–0.692	—	—	—
*τ* ^2^	0.650	—	—	—

Parameter estimates for a regression of the rates of change in annual skeletal extension for *Siderastrea siderea* cores collected from the backreef (“FR”; *n* = 3), nearshore reefs (“NS”; *n* = 3), and forereef (“FR”; *n* = 7). The estimates are for a random intercepts model with slopes varying by reef zone and in which the residuals were assumed to follow an ARMA(1, 1) process. *σ* ^2^ is the residual variance, *ϕ*_1_ is the autoregressive parameter, *θ*_1_ is the moving average parameter, and *τ* ^2^ is the variance of the random intercepts. Notation follows that shown in equation (1).

Although rates of change in annual skeletal extension of nearshore and forereef colonies were more negative when compared with backreef corals, only the forereef colonies exhibited a rate of change in annual skeletal extension that was significantly different from zero (*t* = −3.75, *p*<0.001) over approximately the last century. The rates of change in annual skeletal extension were not significantly different from zero for either backreef (*t* = 1.65, *p* = 0.098) or nearshore (*t* = –1.20, *p* = 0.231) *S. siderea* colonies over this interval (Table 3; Fig. 5).

**Table 3:** Model-derived estimates of the rates of annual skeletal extension (mm yr^-1^ per year) over approximately the last century for each reef zone.

Reefzone	Estimate	SE	*t-*statistic	*p-*value
Backreef (BR)	0.011	0.006	1.65	0.098
Nearshore (NS)	−0.008	0.006	−1.20	0.231
Forereef (FR)	−0.020	0.005	−3.75	<0.001

Results of a random intercepts model in which the residuals were assumed to follow an ARMA(1, 1) process. Displayed are estimated trends in annual skeletal extension over approximately the last century for *Siderastrea siderea* cores collected from the backreef (*n* = 3), nearshore reefs (*n* = 3), and forereef (*n* = 7) study sites.

**Figure 6:**
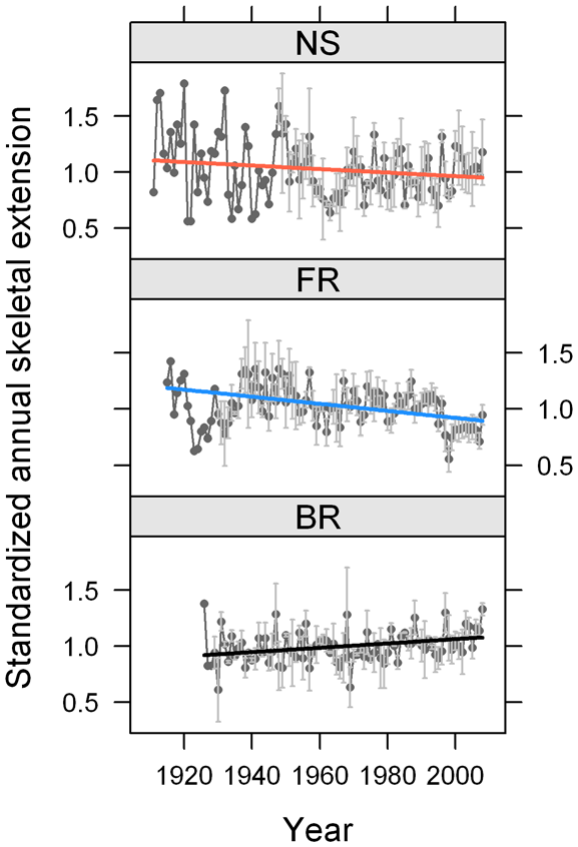
Master chronologies of standardized annual skeletal extension. Master chronologies for forereef (FR), backreef (BR), and nearshore reef (NS) environments with trend lines obtained using generalized least squares for individual core time series, assuming an ARMA (1, 1) correlation structure for the errors. Trend lines are estimated using standardized extension rates as the response. Random intercepts were not necessary in the model due to standardization. Values are means for all cores within each reef zone ±1 standard error when *n*>1. Points based on a single core value have no error bar displayed.

Master chronologies (Fig. 6) compiled for cores from each of the three reef zones also suggest a decrease in standardized annual skeletal extension for forereef *S. siderea,* relative to nearshore and backreef colonies over the studied interval.

## Discussion

### Potential differences in susceptibility and resilience of *Siderastrea siderea* across reef zones

The decline in skeletal extension for forereef *S. siderea* and the relative stability of skeletal extension for backreef and nearshore colonies over the past century suggest that forereef *S. siderea* colonies may be more susceptible to natural and/or anthropogenic stress than backreef and nearshore conspecifics. These differences in susceptibility to natural and/or anthropogenic stress may arise, in part, from differences in the coral colonies' history of exposure to baseline environmental stress amongst the reef zones. This is consistent with previous studies that have shown that exposure to greater stress may increase a coral's resistance to future stressors [28], [45]–[47]. For example, off the coast of Phuket, Thailand, the western sides of *Goniastrea aspera* corals bleached in early 1995 during an interval of high solar radiation. However, later that year, only the eastern sides of these coral colonies bleached when solar radiation was low and higher than normal seawater temperature was the dominant stressor. Bleaching of the eastern sides of these corals and not the western sides during anomalously high seawater temperatures was attributed to pre-conditioning of the western sides during the earlier solar bleaching event [47].

Coral skeletal extension reflects changes in environmental conditions [48]. Thus, the observation that skeletal extension remained unchanged over approximately the last century for *S. siderea* within the historically high-stress nearshore reef environment (subject to increased sedimentation, pollution, and freshwater input) and within the more thermally variable and heat-stressed backreef environment (where restricted circulation supports elevated seawater temperatures), yet decreased for corals from the cooler and more thermally stable forereef environment, suggests that acclimatization [28] and/or adaptation [30], [31] may be important processes influencing the response of these corals to global climate change and increasing anthropogenic stress.

Although *S. siderea* growth trends in the present study support this hypothesis, there are important caveats to consider. First, because all three reef zones are located within marine protected areas, the number of *S. siderea* cores collected from each reef zone was relatively low (forereef  =  7, backreef  =  3, nearshore  =  3). Thus, sample size should be considered in the interpretation of these results. Yet despite the limited number of cores obtained, skeletal growth trends within each reef zone were relatively consistent amongst cores (Fig. 4.), which suggests that the analyzed cores are indeed representative of the greater population of *S. siderea* inhabiting the respective reef zones.

Second, there is evidence that forereef habitats along the southern terminus of the MBRS may be exposed to greater nutrient, sediment, and pollution from the larger and more populated watersheds of Honduras and Guatemala [49]. Indeed, a recent study found that skeletal growth for *Montastraea faveolata* were suppressed longer for colonies closer to the Guatemala and Honduras coast than for those farther away from these coasts [19]. Nevertheless, the findings of this prior study concur with those of the present study in that they both observed a decline in the rate of skeletal extension for forereef corals over recent decades.

Constraining the cause(s) of this decline poses some interesting challenges. In particular, there are numerous confounding factors associated with global climate change and increased anthropogenic stress that must be considered in order to better understand the ecophysiological and/or genetic basis for differential stress responses amongst corals inhabiting different reef zones of the MBRS. These factors will be briefly explored in the sections that follow.

### Potential causes of the differences in rates of change in annual skeletal extension over time

Several confounding factors may contribute to the observed differences in the rate of change in annual skeletal extension amongst *S. siderea* inhabiting the three reef zones of the MBRS [i.e., forereef (strongly negative) < nearshore reef (weakly negative) < backreef (weakly positive)]: (1) increasing seawater temperature resulting from global warming; (2) differences in light attenuation; (3) changing hydraulic regime; (4) increasing sedimentation; (5) eutrophication; (6) increasing pollution; and (7) the combined effects of multiple stressors.

#### Increasing seawater temperature and thermal stress

Seawater temperature differs markedly amongst forereef, backreef, and nearshore reef environments within the MBRS. Backreef and nearshore reefs are generally exposed to warmer and more variable thermal conditions than forereef habitats [28], [50], and these differences in seawater temperature have direct physiological consequences for corals inhabiting each reef zone [28]. The unique temporal patterns of skeletal extension exhibited by *S. siderea* across this inshore-offshore gradient over the past century may have arisen either from differing temperature regimes amongst these three reef zones and/or from differences in the corals' ability to tolerate thermal stress amongst these three reef zones.

Several recent studies have examined the effects of rising seawater temperature and associated thermal stress on coral skeletal extension [15], [21], [22]. In one study, mean annual seawater temperature and annual skeletal extension for *Porites spp.* were examined between 1988 and 2003 on the Great Barrier Reef [22]. Average annual skeletal extension was found to have declined from 1.52 cm yr^−1^ to 1.28 cm yr^−1^, equivalent to a decline of 0.016 cm yr^−1^ per year. The observed decline in skeletal extension was accompanied by an increase in SST over the studied interval. Another recent study compared skeletal extension of *P. lutea* from eight sites off South Thailand between December 1984 and November 1986 and between December 2003 and November 2005 [21]. A comparison of annual skeletal extension with regional SST revealed that rising seawater temperature resulted in a reduction in skeletal extension of 46% to 56% for every 1°C rise in SST. Other studies also report that increasing SST and associated thermal stress have negatively impacted Caribbean corals in recent decades [51], [52].

Annual skeletal extension of the species *M. annularis* has also been reported to decrease with increasing SST in both the Gulf of Mexico (lower average SST) and the Caribbean Sea (higher average SST) [18]. Critically, however, it was observed that this decline was only statistically significant for the Gulf of Mexico corals that were adapted to colder waters, and not for the Caribbean corals that were adapted to warmer waters. This is consistent with the observation of the present study that rates of change in annual skeletal extension of *S. siderea* corals inhabiting the cooler forereef waters of the MBRS were more negative than for corals inhabiting the warmer nearshore reef and backreef zones (Figs. 4, 5, 6). It may be that regular exposure of the backreef and nearshore *S. siderea* colonies to higher maximum seawater temperatures, as well as to more variable temperatures (diurnal, seasonal, and year-to-year) [28], has preconditioned these corals to be more resistant and/or resilient than their forereef counterparts to thermal stress associated with global warming. This suggests that nearshore and backreef *S. siderea* may be more acclimatized and/or adapted to future warming, as well. Conversely, forereef corals, which typically experience cooler more stable diurnal, seasonal, and annual temperature patterns [28] (i.e., more stenothermal environments), may be less conditioned to thermal stress and, therefore, more susceptible than their nearshore and backreef counterparts to future ocean warming.

However, not all studies have revealed that coral skeletal extension is negatively impacted by rising seawater temperatures. A study performed on the fringing reefs of the central Caribbean coast of Panama found that annual skeletal extension for *S. siderea* was not correlated with measured environmental variables, including seawater temperature [25]. A later study performed along the same reef system off the coast of Panama also found that declining skeletal extension for *S. siderea* was not correlated with SST [13]. Although these studies did not identify a relationship between seawater temperature and skeletal extension for *S. siderea*, it is possible that the effect of seawater temperature was masked by the corals' response to other anthropogenic stressors induced by the construction of the Panama Canal (e.g., increased runoff and sedimentation) [13]. In contrast, *S. siderea* colonies near the southern coast of Belize are exposed to significantly less anthropogenic stress [49] due to Belize's relatively small population, smaller watershed sizes, and more limited land clearing and coastal development. Therefore, the effect of seawater temperature on the skeletal extension of *S. siderea* along the sparsely-populated Belize coast may be more obvious than for corals inhabiting areas exposed to greater land-derived anthropogenic stressors.

In addition to rising baseline seawater temperatures within the three reef zones, short-lived fluctuations in temperature may also influence annual skeletal extension amongst the three reef zones of the MBRS. Due to recent global warming, reef corals have been exposed to more frequent and more intense short-lived thermal stress events [1], [53]. Seawater temperatures 1–2°C above the mean monthly summer maximum, for even a brief interval, are known to have negative physiological consequences for corals (e.g., thermally-induced bleaching) [53]–[55]. Coral bleaching results from the reduction in pigment concentration and/or the expulsion of unicellular symbiotic algae (Genus: *Symbiodinium*) from the coral host [54]. In the present study, none of the thirteen *S. siderea* cores examined exhibited evidence of tissue necrosis (e.g., manifest as scarring in the core profile; [19]) throughout the studied growth interval, although we did observe suppression of growth during years when widespread bleaching occurred in the Caribbean (e.g., 1995, 1998, 2005). It should be noted here that thermally driven bleaching events are marked by mortality scars within *M. faveolata* cores obtained from the same general region [19], which highlights potentially fundamental differences in the susceptibility of *S. siderea* and *M. faveolata* to thermal stress.

#### Light

Light levels are also known to affect skeletal extension in corals [56], [57]. In general, coral skeletal extension is positively correlated with light availability due to enhanced photosynthesis, which provides energy for calcification [17], [48], [56]. Light levels along the Belize coast should increase seaward from nearshore to forereef environments mainly due to decreases in the concentration of terrestrially derived sediments [58] and possibly planktonic algae [29]. However, in the southern portion of the MBRS light gradients may be seasonally disrupted or even reversed due to significant input of sediment and nutrients from the larger and more populated watersheds of Guatemala and Honduras [49]. If light intensity increases from the nearshore to the forereef environments, then light attenuation would appear not to be the primary factor driving the differential extension rates amongst *S. siderea* inhabiting the three reef zones. However, if the gradient is reversed due to sediment flux from Guatemala and Honduras, then the observed trends in skeletal extension for nearshore, backreef, and forereef *S. siderea* (i.e., forereef < nearshore < backreef) would be consistent with differences in light availability amongst these three reef zones. Personal observation by one of the authors (albeit anecdotal) of the studied portion of the MBRS suggest that visibility and related light availability for corals is generally higher in the forereef and backreef environments relative to the nearshore environment, except for short intervals following major storm events when the forereef receives substantially more fluvial input from the watersheds of Guatemala and Honduras. Long-term in situ light monitoring along transects across the southern MBRS is needed to resolve this uncertainty.

#### Hydraulic regime

Hydraulic regime has also been shown to affect coral skeletal extension [57]. In eleven reef sites located within 50 km of southern Thailand, *P. lutea* colonies growing in high wave energy regions had lower skeletal extension than those inhabiting lower wave energy reef environments [57]. In the present study, rates of change in skeletal extension were also lower in the generally higher energy shallow forereef environment than in the generally lower energy nearshore and backreef environments. Therefore, we cannot exclude water motion and wave activity as a factor influencing differences in skeletal extension amongst these three reef zones. However, the nearshore and backreef environments of the MBRS sometimes experience wave energies that are either comparable to or even greater than those experienced by forereef environments. Long-term monitoring of hydraulic energy across the southern MBRS is needed to resolve its potential effects on coral skeletal extension amongst the three reef zones.

#### Increased sedimentation

A recent study conducted on *M. faveolata* from the MBRS found that rates of skeletal extension for corals at southern sites remained suppressed for longer intervals of time following stress events than for corals at northern sites [19]. In that study, sedimentation was identified as one of the key factors responsible for the differential growth response of *M. faveolata* from northern and southern localities of the MBRS.

On the Belize portion of the MBRS, sedimentation generally decreases seaward throughout most of the year as sediment-laden terrestrial runoff becomes diluted by open-ocean seawater containing less sediment [58]. However, in the Gulf of Honduras near the southern terminus of the MBRS, this inshore-offshore gradient becomes more complex as the north-south trending coastline of Belize bends at a near right angle to form the coastlines of Guatemala and Honduras. Thus, the southern portions of the MBRS receive sediment from two general directions: eastward-flowing run-off from the sparsely populated watersheds of Belize, and northward-flowing run-off from the larger and more densely populated watersheds of Honduras and Guatemala [49]. Indeed, *Sea-viewing Wide Field-of-view Sensor* (*SeaWIFS*) images and model outputs suggest that most of the terrestrial sediments delivered to the MBRS originate from Guatemala and Honduras [49], [59]. Therefore, the observation in the present study that forereef *S. siderea* exhibit more negative trend in annual skeletal extension than backreef corals (less sedimented because of their distance from the Belize coast and their isolation from the Honduras and Guatemalan coast via the reef crest) over the past century, is consistent with sedimentation being a factor differentiating skeletal growth patterns for this species amongst reef zones. This relationship between sedimentation and skeletal extension for *S. siderea* is consistent with earlier work on *M. faveolata* colonies on the southern MBRS [19], as well as with other studies [60]. However, these observations contrast with a previous study showing that skeletal extension within *S. siderea* across a 30-year interval was not affected by increased sedimentation resulting from urbanization and river discharge along the coast of Puerto Rico [3].

Although sedimentation can result in reduced skeletal extension for some coral species [60], and may have contributed to differences in skeletal extension trends for *S. siderea* amongst the three reef zones investigated in the present study, it is unclear whether sedimentation was the primary factor responsible for these observed differences. Corroboration of modeled sediment outputs with long-term in situ instrumental monitoring of sedimentation across the Belize, Guatemala, and Honduras portions of the MBRS are needed to fully assess the role that increased sedimentation plays in suppressing skeletal extension rates of corals within the MBRS.

#### Eutrophication

Experiments have shown that skeletal extension within zooxanthellate corals can be enhanced by nutrient input [62]. Since nutrient concentrations are known to vary across reef zones [61], eutrophication may be a factor contributing to the differential extension patterns of *S. siderea* amongst the reef zones. Model outputs of terrestrial runoff into the Gulf of Honduras suggest that buoyant matter concentrations (which include dissolved nutrient such as nitrogen and phosphorus [63]) are consistently higher and less variable for nearshore waters along the coast of the MBRS than for more distal backreef and forereef environments [63]. However, within the southern region of the MBRS, this gradient may be reversed, since over three-quarters of all nutrients entering the north-south trending southern MBRS originates from the east-west trending coastlines of Honduras and Guatemala [49]—with greater input during major storm events [59]. This should focus the impact of nutrients from Honduras and Guatemala on the more proximal forereef environment of the southern MBRS, rather than on the more isolated and distal backreef environment. However, we observed declining skeletal extension in forereef *S. siderea*, suggesting that increased nutrients from the watershed of Guatemala and Honduras have not positively influenced skeletal extension for this species.

It has also been shown that in regions where eutrophication and increased sedimentation co-occur, such as along the forereefs of the southern MBRS, nutrient enrichment may enhance photosynthesis within coral symbionts to compensate for the reduced light associated with increased sedimentation [62], [64]. However, forereef *S. siderea* colonies exhibited the greatest decline in skeletal extension over the last century (versus the backreef and nearshore colonies), suggesting that eutrophication did not substantially mitigate the effects of increased sedimentation and/or that other environmental stressors, such as increasing seawater temperatures, may have had a greater impact on skeletal extension patterns for this species.

Eutrophication is also known to negatively affect coral skeletal extension by promoting the growth of algae, which prevent light and food from reaching the coral and which compete with corals for space on the reef [65]. This negative effect of eutrophication may be a possible explanation for the observed decrease in skeletal extension of *S. siderea* inhabiting the forereef environment of the southern MBRS.

#### Pollution

Pollution may also have contributed to the observed differences in skeletal extension amongst the three reef zones. Our results are consistent with the expectation that forereef *S. siderea* colonies, which are more proximal to the larger and more densely populated watersheds of the Honduran and Guatemalan coast—the primary source of the pollution to this region [63]—was more negatively impacted than colonies on the nearshore and backreef environments that are more proximal to the sparsely populated coast of southern Belize. These results are consistent with a recent study of *M. faveolata* cores obtained along a north-south transect of the MBRS, which reported that corals bordering the Sapodilla Cayes (Fig. 1) experienced the greatest impact of terrestrial runoff, as inferred from Ba/Ca measurements of the corals' skeletons [66]. It is therefore conceivable that the lower skeletal extension for forereef *S. siderea*, compared with backreef and nearshore *S. siderea*, was due to pollution derived from river effluents originating from Guatemala and Honduras [59]. However, prior studies report that skeletal extension for *S. siderea* [13], as well as for other reef-building corals off the coast of Indonesia [17], have not been materially affected by various land-derived pollutants.

#### The compounding effects of multiple stressors

The compounding effects of multiple stressors on coral skeletal extension may be more important than the effect of any single stressor [19], [66]. In a recent study on the MBRS, skeletal extension of *M. faveolata* over approximately the last century was investigated to determine whether local stressors reduce the thermal tolerance of corals. This study found that the combination of chronic local stressors (represented by increasing human population) and temperature was a better predictor of coral skeletal extension than temperature alone. A companion study constructed century-scale records of trace-metal/calcium ratios (proxies of local environmental stress) over time for *M. faveolata* cores collected from sites along the MBRS [66]. The results indicated that local stressors on the MBRS have increased steadily over time and that these stressors were higher in the southern part of the reef system than in the northern part. Model outputs suggest that anthropogenic alteration of landscapes bordering the Gulf of Honduras has caused the increased erosion, runoff, and nutrient delivery evident in the southern portion of the MBRS [49]. These results provide compelling evidence that anthropogenic stress from the continent is a major factor responsible for the more drastic decline in skeletal extension observed for *M. faveolata* in the southern portions of the MBRS, compared to the northern portions.

It is plausible that the decline in skeletal extension for forereef *S. siderea* observed in the present study resulted from a combination of anthropogenic stressors, similar to that which is thought to have caused the decline in skeletal extension for *M. faveolata* in this region over a similar temporal interval [67]. This could possibly explain the relative stability in skeletal extension for backreef and nearshore *S. siderea*, which were generally exposed to lower anthropogenic stress because of their proximity to the less-densely populated southern Belize coast over this interval. However, it is also possible that historically greater baseline levels of environmental stress (i.e., non-anthropogenic) within the backreef and nearshore environments of the MBRS may have pre-conditioned *S. siderea* corals within these reef zones, effectively increasing their resistance/resilience to recent anthropogenic stress. However, as industrialization proceeds and global populations continue to expand, corals will be exposed to even greater anthropogenic stress (temperature, sedimentation, eutrophication, pollution, etc.). Skeletal extension within the apparently more resistant/resilient backreef and nearshore *S. siderea* colonies of the southern MBRS may commence a more precipitous decline once their stress-tolerance thresholds are exceeded.

### Conclusion

Rates of change in annual skeletal extension over the past century for forereef *S. siderea* corals of the MBRS have been more negative than for nearshore corals, which have been more negative than for backreef corals. However, only rates of change in annual skeletal extension for forereef *S. siderea* (negative) were significantly different from zero; rates of changes in annual skeletal extension for nearshore and backreef *S. siderea* corals were not significantly different from zero. Furthermore, since the early 1900s, average annual skeletal extension for backreef *S. siderea* colonies has been consistently higher than for nearshore colonies, while forereef colonies have transitioned from exhibiting the fastest to the slowest average annual skeletal extension.

The reasons that rates of change in annual skeletal extension of *S. siderea* corals differ amongst these three reef zones over the studied interval are not well constrained by the available data. However, differential thermal stress (resulting from global warming) and/or differential acclimation/adaptation to thermal stress amongst the three zones may be driving these disparate trends amongst reef zones. Increasing local anthropogenic stress (sedimentation, eutrophication, pollution, and other factors not discussed here such as ocean acidification and rising sea level) may also contribute to the differential responses of *S. siderea* amongst these three reef zones, especially as these stressors (and the negative response of the corals) appear to be greatest for forereef environments that are more proximal to the larger and more densely populated coastal watersheds of Honduras and Guatemala. It should also be noted here that these putative relationships between skeletal extension and natural/anthropogenic stressors may be species dependent. *S. siderea* is known to be a particularly hardy species that is able to survive harsh environmental conditions [25]. Therefore, temporal trends in skeletal extension for this species may differ from those for other coral species, even under similar ambient conditions.

The results presented here suggest that forereef colonies of *S. siderea* within the Gulf of Honduras may be more susceptible to environmental and anthropogenic stress, including future ocean warming, than their backreef and nearshore counterparts. Coral reef managers within the MBRS should consider potential differences in the sensitivity of reef zones when allocating resources to the protection and maintenance of reef ecosystems. Additional research is required to investigate the precise nature of the relationship between environmental and anthropogenic stressors and skeletal extension of *S. siderea* across the various reef zones of the MBRS, and to determine whether the pattern observed in the present study is exhibited by other species of reef-building scleractinian corals within the MBRS, and within other reef systems around the world.

## Supporting Information

Table S1 Population of countries along the Mesoamerican Barrier Reef System, excluding Mexico.
(0.03 MB DOC)
