# Supplementary material for: Declining Coral Skeletal Extension for Forereef Colonies of Siderastrea siderea on the Mesoamerican Barrier Reef System, Southern Belize
Source: PLoS One. 2011 Feb 16;6(2):e14615. doi: 10.1371/journal.pone.0014615 (PMC3040180; doi:10.1371/journal.pone.0014615)
Supplement: Table S1 — Population of countries along the Mesoamerican Barrier Reef System, excluding Mexico. (0.03 MB DOC) [file pone.0014615.s001.doc]

**Table S1. Population of countries along the Mesoamerican Barrier Reef System, excluding Mexico**

| **Country along the Mesoamerican Barrier Reef** | **Population 1990 (Millions)** | **Population**  **2000 (Millions)** | **Population mid-2010 (Millions)** | **Population in watershed draining into the Caribbean 2000 (Millions)** | **Estimate of population in watershed draining into the Caribbean mid-2010 (Millions)** |
| --- | --- | --- | --- | --- | --- |
| Belize | 0.19 | 0.26 | 0.35 | 0.26 | 0.35 |
| Guatemala | 8.75 | 11.38 | 14.4 | 6.20 | 7.87 |
| Honduras | 4.87 | 6.42 | 7.60 | 4.27 | 5.05 |
| Total | 13.81 | 18.03 | 22.35 | 10.73 | 13.30 |

Source of 1990 and 2000 population data (Burke and Maidens 2004). Mid-2010 population data were acquired from the Population Reference Bureau, World Population Datasheet 2010 (<http://www.prb.org/Publications/Datasheets/2010/2010wpds.aspx>). Population within watershed drainage area for year 2000 were also taken from Burke and Maidens (2004). Mid-2010 population data for watersheds draining into the Caribbean are estimates based on the change in population between 2000 and mid-2010.
